# Supplementary material for: Feasibility, acceptability and validation of wearable devices for climate change and health research in the low-resource contexts of Burkina Faso and Kenya: Study protocol
Source: PLoS One. 2021 Sep 30;16(9):e0257170. doi: 10.1371/journal.pone.0257170 (PMC8483291; doi:10.1371/journal.pone.0257170)
Supplement: S1 File — (PDF) [file pone.0257170.s001.pdf]

Generated by cp1, Sep 02, 2021 17:57
Questionnaire created by cp1, Jun 28, 2021 16:47
Last modified by dobor, Aug 12, 2021 10:44

Shared with:
qollinsochieng last edited 8/6/2021 5:22:19 PM
dobor last edited 8/12/2021 8:44:07 AM

Sections: 4, Sub-sections: 0, Questions: 73.
Questions with enabling conditions: 14
Questions with validation conditions:1
Rosters: 2
Variables: 0

Survey Solutions

CP1 Feasibility study

SURVEY IDENTIFICATION INFORMATION
QUESTIONNAIRE DESCRIPTION

STUDY PARTICIPANT'S DETAILS
No sub-sections, No rosters, Questions: 9, Static texts: 1.

DISTRIBUTION/ RETURN OF DEVICE
No sub-sections, Rosters: 2, Questions: 28.

ACCEPTANCE QUESTIONNAIRE
No sub-sections, No rosters, Questions: 29, Static texts: 1.

ACTIVITY DIARY
No sub-sections, No rosters, Questions: 7.

APPENDIX A — CATEGORIES

LEGEND

SURVEY IDENTIFICATION INFORMATION QUESTIONNAIRE DESCRIPTION

Basic information

Title CP1 Feasibility study

Survey data information

Study type Integrated Survey

Kind of data Aggregate data [agg]

Survey information

Country Burkina Faso

Year 2020

Languages French

Funding DFG

STUDY PARTICIPANT'S DETAILS

study_part_details

STATIC TEXT

Study procedure: --- 1. Take the participant's device --- --- 2. Disinfect the participant's device --- --- 3. Put the device on the charger --- --- 4. Synchronize the data of the device with the study participant's profile by using the app on the tablet --- --- 5. Fill in the section ""Distribution/return of device"" --- --- 6. Take the participant's blood pressure measurement and record it in the Health Mate application --- --- 7. Ask the questions of the acceptance questionnaire --- --- 8. Ask the questions of the activity diary questionnaire ---

Name of participant's village	SINGLE-SELECT: COMBO BOX — nom_village<br>001 ○ Barakuy<br>002 ○ Toni<br>003 ○ Biron Bobo<br>004 ○ Biron Marka<br>005 ○ Boron<br>006 ○ Bouni/Boune<br>007 ○ Bourasso<br>008 ○ Cisse<br>009 ○ Dankoumana<br>010 ○ Dembelela<br>011 ○ Denissa<br>012 ○ Denissa Mossi<br>013 ○ Diamasso<br>014 ○ Dionkongo<br>015 ○ Dina<br>016 ○ Dokoura<br><br>And 272 other symbols [4]
Location ID of participant's HDSS residence	TEXT — locationid<br>..
Household ID	TEXT — idhh<br>..
Full name of study participant	TEXT — study_part_name<br>..
Enter the participant's study ID<br><br>I L'identifiant du participant est de 14 position .<br>V1 `Regex.IsMatch(self,"^\\d{1,3}-\\d{1,3}-\\d{1,3}-\\d{1,3}$") || Regex.IsMatch(self,"Q")`	TEXT — study_part_id<br>..
Name of the Compound head	TEXT — compheadname<br>..
Gender of participant	SINGLE-SELECT — sex<br>01 ○ Male<br>02 ○ Female
ID of household head	TEXT — hh_head_id<br>..

Name of Household head	TEXT<br>..	hh_head_name

DISTRIBUTION/ RETURN OF DEVICE

sensor_exchange

Would you like to distribute or to return a device?	SINGLE-SELECT return_handout_sensor<br>01 ○ Distribution - I would like to start the distribution of a device.<br>02 ○ Return - I would like to start the return of a device.
Which device has been distributed?<br>E return_handout_sensor==1	MULTI-SELECT sensor_type_distrib<br>01 ☐ Withings Pulse HR (black wristband)<br>02 ☐ Tucky thermometer (white patch)<br>03 ☐ Neither

DISTRIBUTION/ RETURN OF DEVICE

Roster: WHICH DEVICE HAS BEEN DISTRIBUTED? - %ROSTERTITLE%

generated by multi-select question sensor_type_distrib roster_1_sensor_distributed

E return_handout_sensor==1

Which number has the distributed device %rostertitle%?	NUMERIC: INTEGER num_sensor_distributed<br>_ .
Please note the distribution date of the device %rostertitle%.<br>I Please fill in the date the following way: day (dd) - month (mm) - year (y y)	DATE sensor_date_distributed<br>..
Is the device %rostertitle% disinfected?	SINGLE-SELECT sensor_desinfected<br>01 ○ Yes, it is disinfected.<br>02 ○ No, it isn't disinfected.<br>03 ○ Comment
Comment (disinfection of the device)<br>E sensor_desinfected==3	TEXT sensor_desinfected_comm<br>..
The study participant is registered in the app on the tablet for the device %rostertitle%?	SINGLE-SELECT registration_stud_part_app<br>01 ○ Yes, I have registered the study participant in the app.<br>02 ○ No, I haven't registered the study participant in the app.<br>03 ○ Comment
Comment (registration)<br>E registration_stud_part_app==3	TEXT registration_stud_part_app_comm<br>..
Are you comfortable with the device's %rostertitle% tablet application?	SINGLE-SELECT ease_app_distribution<br>01 ○ Strongly agree<br>02 ○ Agree<br>03 ○ Neither agree nor disagree<br>04 ○ Disagree<br>05 ○ Strongly disagree

Has it been easy for you to synchronize the data of the device with the study participant?	SINGLE-SELECT difficulty_usage_app_distributi<br>01 ◯ Strongly agree<br>02 ◯ Agree<br>03 ◯ Neither agree nor disagree<br>04 ◯ Disagree<br>05 ◯ Strongly disagree
It has been easy for you to manage the study participant with the tablet.	SINGLE-SELECT knowledge_level_app_distribution<br>01 ◯ Strongly agree<br>02 ◯ Agree<br>03 ◯ Neither agree nor disagree<br>04 ◯ Disagree<br>05 ◯ Strongly disagree
The participant is satisfied with the device %rostertitle%.	SINGLE-SELECT feedback_stud_part_distribution<br>01 ◯ Strongly agree<br>02 ◯ Agree<br>03 ◯ Neither agree nor disagree<br>04 ◯ Disagree<br>05 ◯ Strongly disagree
The participant accepted the device %rostertitle%.	SINGLE-SELECT acceptance_stud_part_distributio<br>01 ◯ Strongly agree<br>02 ◯ Agree<br>03 ◯ Neither agree nor disagree<br>04 ◯ Disagree<br>05 ◯ Strongly disagree
The participant was happy to wear the device %rostertitle%.	SINGLE-SELECT happy_stud_part_distribution<br>01 ◯ Strongly agree<br>02 ◯ Agree<br>03 ◯ Neither agree nor disagree<br>04 ◯ Disagree<br>05 ◯ Strongly disagree
The participant is frustrated by the device %rostertitle%.	SINGLE-SELECT frustration_stud_part_distributi<br>01 ◯ Strongly agree<br>02 ◯ Agree<br>03 ◯ Neither agree nor disagree<br>04 ◯ Disagree<br>05 ◯ Strongly disagree
Which device has been returned?<br>E return_handout_sensor==2	MULTI-SELECT sensor_type_returned<br>01 ☐ Withings Pulse HR (black wristband)<br>02 ☐ Tucky thermometer (white patch)<br>03 ☐ Neither

DISTRIBUTION/ RETURN OF DEVICE

Roster: WHICH DEVICE HAS BEEN RETURNED? - %ROSTERTITLE%

generated by multi-select question sensor_type_returned roster_2_sensor_returned

E return_handout_sensor==2

Which number has the returned device %rostertitle%?	NUMERIC: INTEGER num_sensor_returned
Please enter the return date of the device %rostertitle%.<br>I Please fill in the date the following way: day (dd) - month (mm) - year (y y)	DATE sensor_date_returned
Is the device defect?	SINGLE-SELECT sensor_status_returned<br>01 Yes, it is defect.<br>02 No, it works.<br>03 Other
Other (device %rostertitle% defect)<br>E sensor_status_returned==3	TEXT comment_sensor_status_returned
Are you comfortable with the device's %rostertitle% tablet application?	SINGLE-SELECT ease_usage_app<br>01 Strongly agree<br>02 Agree<br>03 Neither agree nor disagree<br>04 Disagree<br>05 Strongly disagree
It has been easy for you to synchronize the data of the study participant's device.	SINGLE-SELECT difficulty_usage_app<br>01 Strongly agree<br>02 Agree<br>03 Neither agree nor disagree<br>04 Disagree<br>05 Strongly disagree
It has been easy for you to manage the study participant with the tablet.	SINGLE-SELECT knowledge_level_app<br>01 Strongly agree<br>02 Agree<br>03 Neither agree nor disagree<br>04 Disagree<br>05 Strongly disagree
The participant is satisfied with the device %rostertitle%.	SINGLE-SELECT feedback_stud_part<br>01 Strongly agree<br>02 Agree<br>03 Neither agree nor disagree<br>04 Disagree<br>05 Strongly disagree
The participant accepted the device %rostertitle%.	SINGLE-SELECT acceptance_stud_part<br>01 Strongly agree<br>02 Agree<br>03 Neither agree nor disagree<br>04 Disagree<br>05 Strongly disagree

The participant was happy to wear the device %rostertitle%.	SINGLE-SELECT happy_stud_part<br>01 ○ Strongly agree<br>02 ○ Agree<br>03 ○ Neither agree nor disagree<br>04 ○ Disagree<br>05 ○ Strongly disagree
The participant is frustrated by the device %rostertitle%.	SINGLE-SELECT frustration_stud_part<br>01 ○ Strongly agree<br>02 ○ Agree<br>03 ○ Neither agree nor disagree<br>04 ○ Disagree<br>05 ○ Strongly disagree
Other comments	TEXT general_comments_sensor_return<br>..

ACCEPTANCE QUESTIONNAIRE

STATIC TEXT

Before starting the interview with the study participant, please put the (s) device (s) on the charging station. Please ask the study participant all of the questions. Thank you for your accurate collaboration.

I am happy to use the device.	SINGLE-SELECT positive_att_sensor<br>01 ○ Strongly agree<br>02 ○ Agree<br>03 ○ Neither agree nor disagree<br>04 ○ Disagree<br>05 ○ Strongly disagree
I am upset using the device	SINGLE-SELECT disturbed_sensor<br>01 ○ Strongly agree<br>02 ○ Agree<br>03 ○ Neither agree nor disagree<br>04 ○ Disagree<br>05 ○ Strongly disagree
Are you wearing more than one device?	SINGLE-SELECT multiple_sensors<br>01 ○ Yes<br>02 ○ No
I felt comfortable with multiple devices.<br>E `multiple_sensors == 1`	SINGLE-SELECT experience1_multiple_sensors<br>01 ○ Strongly agree<br>02 ○ Agree<br>03 ○ Neither agree nor disagree<br>04 ○ Disagree<br>05 ○ Strongly disagree
It has been difficult for me to wear multiple devices.<br>E `multiple_sensors==1`	SINGLE-SELECT experience2_multiple_sensors<br>01 ○ Strongly agree<br>02 ○ Agree<br>03 ○ Neither agree nor disagree<br>04 ○ Disagree<br>05 ○ Strongly disagree
It has been uncomfortable for me to wear multiple devices.<br>E `multiple_sensors==1`	SINGLE-SELECT experience3_multiple_sensors<br>01 ○ Strongly agree<br>02 ○ Agree<br>03 ○ Neither agree nor disagree<br>04 ○ Disagree<br>05 ○ Strongly disagree
I liked wearing multiple devices.<br>E `multiple_sensors==1`	SINGLE-SELECT experience4_multiple_sensors<br>01 ○ Strongly agree<br>02 ○ Agree<br>03 ○ Neither agree nor disagree<br>04 ○ Disagree<br>05 ○ Strongly disagree

It has been easy for me to wear the device this week.	SINGLE-SELECT week_experience1_sensor<br>01 ○ Strongly agree<br>02 ○ Agree<br>03 ○ Neither agree nor disagree<br>04 ○ Disagree<br>05 ○ Strongly disagree
It has been uncomfortable for me to wear the device this week.	SINGLE-SELECT week_experience2_sensor<br>01 ○ Strongly agree<br>02 ○ Agree<br>03 ○ Neither agree nor disagree<br>04 ○ Disagree<br>05 ○ Strongly disagree
What have you liked/ disliked about the device? (multiple choice)	MULTI-SELECT: ORDERED like_dislike_sensor<br>01 ☐ easy to wear<br>02 ☐ good weight<br>03 ☐ good handiness<br>04 ☐ nice appearance<br>05 ☐ practical to wear<br>06 ☐ comfortable to wear<br>07 ☐ too bulky<br>08 ☐ too heavy<br>09 ☐ too big<br>10 ☐ difficult to wear<br>11 ☐ Other (please describe)
Comment (like/ dislike) - please describe	TEXT like_dislike_sensor_comment<br>..
Did you have any problems with the device this week?	SINGLE-SELECT challenges_sensor<br>01 ○ Yes<br>02 ○ No
This week's problems with the device. (multiple choice)<br>E challenges_sensor==1	MULTI-SELECT: ORDERED challenges_sensor_y<br>01 ☐ itchy skin<br>02 ☐ itch<br>03 ☐ the device caused pain<br>04 ☐ it limited my movements<br>05 ☐ it was disturbing during work<br>06 ☐ it was disturbing during sleep<br>07 ☐ it was disturbing the daily routine<br>08 ☐ electric shock<br>09 ☐ Other (please describe)
Comment (this week's problems with the device) - please describe	TEXT challenges_sensor_yes_comments<br>..
Wearing the device affected my work/ my daily activities.	SINGLE-SELECT daily_life_sensor<br>01 ○ Strongly agree<br>02 ○ Agree<br>03 ○ Neither agree nor disagree<br>04 ○ Disagree<br>05 ○ Strongly disagree

Question	Response
How did it feel to wear the device? (multiple choice)	MULTI-SELECT: ORDERED — reactions_sensor<br>01 ☐ I forgot that I was wearing it.<br>02 ☐ I have not been disturbed.<br>03 ☐ Sometimes difficult<br>04 ☐ it needed time/ attention<br>05 ☐ I have interrupted my activities several times because of the device.<br>06 ☐ I had to remove the device<br>07 ☐ The device limited my movements<br>08 ☐ I had pain or other undesired effects (like a skin rash, itching)<br>09 ☐ The sensor was stuck to my skin because of the sweat.<br>10 ☐ I felt an increase in heat from wearing the sensor.<br>11 ☐ Other (please describe)
Comment (device's effects) - please describe	TEXT — reactions_sensor_comment<br>..
Has wearing the device had effects on your sleep?	SINGLE-SELECT — sleep_sensor<br>01 ○ Yes<br>02 ○ No
Wearing the device had the following effects on my sleep... (multiple choice)<br>E sleep_sensor==1	MULTI-SELECT: ORDERED — sleep_sensor_2<br>01 ☐ I woke up sometimes.<br>02 ☐ I woke up frequently.<br>03 ☐ I couldn't sleep at all because of the device.<br>04 ☐ I felt tired in the morning (poor sleep quality).<br>05 ☐ The device fell off me during the night.<br>06 ☐ I felt an increase in heat from wearing the device.<br>07 ☐ Other (please describe)
Comments (device's effects on my sleep) - please describe	TEXT — sleep_sensor_3<br>..
Did you have to remove the device?	SINGLE-SELECT — removing_sensor<br>01 ○ Yes<br>02 ○ No
I removed the device because... (multiple choice)<br>E removing_sensor==1	MULTI-SELECT: ORDERED, YES/NO — removing_sensor_2<br>01 ☐ / ☐ It was limiting my activities<br>02 ☐ / ☐ I had undesired effects<br>03 ☐ / ☐ Other (please describe)
Comment (removement of the device) - please describe	TEXT — removing_sensor_3<br>..

I was comfortable wearing the device in public	SINGLE-SELECT sensor_public<br>01 ○ Strongly agree<br>02 ○ Agree<br>03 ○ Neither agree nor disagree<br>04 ○ Disagree<br>05 ○ Strongly disagree
Have people asked you about the device?	SINGLE-SELECT sensor_public_2<br>01 ○ Yes<br>02 ○ No
On which of the devices have you been asked questions?<br>E sensor_public_2 == 1	MULTI-SELECT sensor_public_2_1<br>01 ☐ Withings Pulse HR (black wristband)<br>02 ☐ Tucky thermometer (white patch)<br>03 ☐ Neither
Please describe, what people have asked you about the device.<br>E sensor_public_2 == 1	TEXT sensor_public_2_2<br>..
If you had to wear this device for a longer period of time (i.e. one year), what would be the barriers for you to participate in such a study (multiple choice)	MULTI-SELECT longterm_sensor<br>01 ☐ Time required to wear<br>02 ☐ time spent participating in studies<br>03 ☐ the sensor does not give me any information about my health<br>04 ☐ Undesired effects<br>05 ☐ Social acceptance<br>06 ☐ Disturbance of daily activities<br>07 ☐ Disturbance of my sleep<br>08 ☐ Disturbance of my personal hygiene routine<br>09 ☐ Other (please describe)
Comment (barriers of a long-term device study) - please describe	TEXT longterm_sensor_comment<br>..

ACTIVITY DIARY

What activity did you do after you got up in the morning? (multiple choice)	MULTI-SELECT: ORDERED `after_gettingup`<br>01 ☐ working on the farm<br>02 ☐ cooking<br>03 ☐ selling products at the market<br>04 ☐ animal husbandry<br>05 ☐ taking care of children<br>06 ☐ taking care of husband/ wife<br>07 ☐ taking care of family members<br>08 ☐ going to school<br>09 ☐ getting water from the well<br>10 ☐ harvesting<br>11 ☐ working on the field<br>12 ☐ I rested<br>13 ☐ watching TV<br>14 ☐ going to a bar<br>15 ☐ going to a restaurant<br>16 ☐ doing sport<br><br>And 5 other symbols [1]
What activity did you do in the morning? (multiple choice)	MULTI-SELECT `morning_activity`<br>01 ☐ working on the farm<br>02 ☐ cooking<br>03 ☐ selling products at the market<br>04 ☐ animal husbandry<br>05 ☐ taking care of children<br>06 ☐ taking care of husband/ wife<br>07 ☐ taking care of family members<br>08 ☐ going to school<br>09 ☐ getting water from the well<br>10 ☐ harvesting<br>11 ☐ working on the field<br>12 ☐ I rested<br>13 ☐ watching TV<br>14 ☐ going to a bar<br>15 ☐ going to a restaurant<br>16 ☐ doing sport<br><br>And 5 other symbols [1]

What activity did you do in the midday? (multiple choice)	MULTI-SELECT `noon_activity`<br>01 ☐ working on the farm<br>02 ☐ cooking<br>03 ☐ selling products at the market<br>04 ☐ animal husbandry<br>05 ☐ taking care of children<br>06 ☐ taking care of husband/ wife<br>07 ☐ taking care of family members<br>08 ☐ going to school<br>09 ☐ getting water from the well<br>10 ☐ harvesting<br>11 ☐ working on the field<br>12 ☐ I rested<br>13 ☐ watching TV<br>14 ☐ going to a bar<br>15 ☐ going to a restaurant<br>16 ☐ doing sport<br><br>And 5 other symbols [1]
What activity did you do in the afternoon? (multiple choice)	MULTI-SELECT `afternoon_activity`<br>01 ☐ working on the farm<br>02 ☐ cooking<br>03 ☐ selling products at the market<br>04 ☐ animal husbandry<br>05 ☐ taking care of children<br>06 ☐ taking care of husband/ wife<br>07 ☐ taking care of family members<br>08 ☐ going to school<br>09 ☐ getting water from the well<br>10 ☐ harvesting<br>11 ☐ working on the field<br>12 ☐ I rested<br>13 ☐ watching TV<br>14 ☐ going to a bar<br>15 ☐ going to a restaurant<br>16 ☐ doing sport<br><br>And 5 other symbols [1]

What activity did you do in the evening? (multiple choice)	MULTI-SELECT<br>01 ☐ working on the farm<br>02 ☐ cooking<br>03 ☐ selling products at the market<br>04 ☐ animal husbandry<br>05 ☐ taking care of children<br>06 ☐ taking care of husband/ wife<br>07 ☐ taking care of family members<br>08 ☐ going to school<br>09 ☐ getting water from the well<br>10 ☐ harvesting<br>11 ☐ working on the field<br>12 ☐ I rested<br>13 ☐ watching TV<br>14 ☐ going to a bar<br>15 ☐ going to a restaurant<br>16 ☐ doing sport<br><br>And 5 other symbols [1]	evening_activity
What activity did you do in the night? (multiple choice)	MULTI-SELECT<br>01 ☐ working on the farm<br>02 ☐ cooking<br>03 ☐ selling products at the market<br>04 ☐ animal husbandry<br>05 ☐ taking care of children<br>06 ☐ taking care of husband/ wife<br>07 ☐ taking care of family members<br>08 ☐ going to school<br>09 ☐ getting water from the well<br>10 ☐ harvesting<br>11 ☐ working on the field<br>12 ☐ I rested<br>13 ☐ watching TV<br>14 ☐ going to a bar<br>15 ☐ going to a restaurant<br>16 ☐ doing sport<br><br>And 5 other symbols [1]	night_activity
Please add the activity (and the time of the day) and describe it, if possible.	TEXT<br>..	comment_activity

APPENDIX A — CATEGORIES

[1] Categories_Activity_Diary
Categories: 1:working on the farm, 2:cooking, 3:selling products at the market, 4:animal husbandry, 5:taking care of children, 6:taking care of husband/ wife, 7:taking care of family members, 8:going to school, 9:getting water from the well, 10:harvesting, 11:working on the field, 12:I rested, 13:watching TV, 14:going to a bar, 15:going to a restaurant, 16:doing sport, 17:driving a vehicle, 18:housekeeping, 19:I have been shopping, 20:cleaning, 21:working in a sitting position

[2] Categories_LikertScale
Categories: 1:Strongly agree, 2:Agree, 3:Neither agree nor disagree, 4:Disagree, 5:Strongly disagree

[3] Categories_Sensors
Categories: 1:Withings Pulse HR (black wristband), 2:Tucky thermometer (white patch), 3:Neither

[4] nom_village: Name of participant's village
Categories: 1:Barakuy, 2:Toni, 3:Biron Bobo, 4:Biron Marka, 5:Boron, 6:Bouni/Boune, 7:Bourasso, 8:Cisse, 9:Dankoumana, 10:Dembelela, 11:Denissa, 12:Denissa Mossi, 13:Diamasso, 14:Dionkongo, 15:Dina, 16:Dokoura, 17:Goni, 18:Kamadena, 19:Kemena, 20:Kodougou, 21:Koro, 22:Labarani, 23:Lei, 24:Lekuy, 25:Lemini, 26:Nokuy, 27:Ouette, 28:Pa, 29:Sampopo, 30:Seriba, 31:Sien, 32:Sikoro, 33:Sobon, 34:Solimana, 35:Sirakoro/Sirakorosso, 36:Tebere, 37:Tonsere, 38:Zanakuy, 39:Tissi, 40:Dara, 41:Bankoumani1, 43:Babekolon, 44:Bagala, 45:Biron badala, 46:Bissau, 47:Bokuy, 48:Damandigui, 49:Hinkuy, 50:Kamiankoro, 51:Kansara, 52:Kerena, 53:Konkuini, 54:Koredougou, 55:Moinsi, 56:Mourdie, 57:Sere, 58:Soin, 59:Tonkoroni, 60:BABAKUY, 62:BANGASSI-KORO, 63:BANGASSI_KOUROU, 64:BARANI, 65:NOUNA, 66:BOGO3, 67:BOULEMPORO, 68:BOULE, 69:DIAMAHOUN, 70:DIENWELY, 71:DJALLO, 72:DOURE, 73:GNIMANOU, 74:ILLA, 75:KAMANDADOUGOU, 76:KAREKUY3, 77:KESSEKUY, 78:KINSERE, 79:KOLONKAN GOURE BA, 80:KOLONKAN GOURE DIALLO, 81:KONKORO, 82:KORONI, 83:KOUBE, 84:KOULEROU, 85:MANEKUY, 86:MANTAMOU, 87:MEDOUGOU, 88:NABASSO, 89:NIAKO, 91:OUEMBOYE, 92:OUERESSE, 93:PAMPAKUY, 94:SEKUY, 95:SEKUY-IRA, 96:SOKOURA3, 97:SOUDOGO, 98:TIRA, 99:TOROKOTO, 100:WARIBERE, 101:YALANKORO, 102:BANAKORO, 103:BOGO4, 104:BOMBOROKUY, 105:BOREKUY, 106:DANEKUY, 107:GOMBELE, 108:KOMONKUY, 109:MARIASSO, 110:NIANKOUINI, 111:SADIGAN, 112:SAKO, 113:SOUANKUY, 114:TIRAKUY, 115:YABANA, 116:YALLO, 117:BA, 118:BANANA, 119:BANKOUMANA, 120:BARA, 121:BERKOUE, 122:BIDA, 123:BOKORO, 124:BONOUA, 125:BOUAKUY, 126:BOURIO, 127:DIEKUINI, 128:DIENA, 129:DIEKAN, 130:DJIBASSO, 131:DONKORO, 132:FONI-BORONKIN, 133:GNIMINI, 134:IRA, 135:KANSARA, 136:KIENEKUY, 137:KIRA, 138:KIE, 139:KOLONKAN, 140:KOLONKANI-SIRAKORO, 141:KOLONZO5, 142:KOMBORI5, 143:MANDARA, 144:MAOULENA, 145:MASSAKUY, 146:MOUNA, 147:NAIRENA, 148:OUAROKUY, 149:OURA, 150:OUROUKO, 151:PARAKUY, 152:PARANZO, 153:PIA N 1, 154:SABA, 155:SADIGNAKONO, 156:SAKUY, 157:SAMEKUY, 158:SARAKORO, 159:SENOULO, 160:SIEDOUGOU, 161:SOUMOUKUY, 162:SOUNE, 163:SOYE, 164:TIEME, 165:VORO, 166:AYOUBAKOLON, 167:BONIKUY, 168:DAR-ES-SALAM, 169:DASSI, 170:DOKUY, 171:DOUBALE, 172:GASSINGO, 173:ILABEKOLON, 174:KANADOUGOU, 175:KARASSO, 176:KEMENSO, 177:KENEKUY, 178:KOLONIDARA, 179:KOLONKOURA6, 180:MAKUY, 181:NEREKO, 182:SOKOURA6, 183:SOUM, 184:SOUMAKORO, 185:TOMIKORONI, 186:BAMPERLA, 187:BANGASSI-BOBO, 188:BANGASSI-ILLA, 189:BANGASSI-MAMOUDOUD, 190:BANKUY, 191:BASSAM, 192:BOANEKUY, 193:BOKUY7, 194:BOUKUY, 195:DAKUY, 196:DOUMBALA, 197:HENLEKUY, 198:KAREKUY7, 199:KIMBA, 200:KINI-KINI, 201:KOA, 202:KODARA, 203:KOLONZO7, 204:KONKUY-BOHO, 205:KONKUY-KORO, 206:KOURKUY, 207:LANFIERA, 208:MONTIONKUY, 209:MOUNAKORO, 210:NIAN, 211:POROKUY, 212:SAINT-CAMILLE, 213:SAINT-MARTIN, 214:SAINT-PAUL, 215:SAWOROKUY, 216:SIMBORA, 217:TENI, 218:TENI-PEULH, 219:TIOURKUY, 220:WANZAN, 221:ZEKUY, 222:ABAYE, 223:AOUREMA8, 224:BA-PEULH, 225:DAGA, 226:GANI, 227:KOLONKANI-BA, 228:KOMBORI8, 229:KONNA, 230:LONANI, 231:MAGADIAN, 232:OUORI, 233:SANAKADOUGOU, 234:SASSAMBARI, 235:SIEKORO, 236:SIEWALI, 237:SIGUIDE, 238:YARAN, 239:BANKOUMANI, 240:BOKUY9, 241:DINA, 242:KIKO, 243:KOLOKAN, 244:MADOUBA, 245:PIA N 2, 246:PORO, 247:TOUBA, 248:YOUROUNA, 249:AOUREMA1, 250:BARE, 251:DANTIERA, 252:DEMBO, 253:DIGANI, 254:DIONDOUGOU, 255:FARAKUY, 256:KAKI, 257:KALFADOUGOU, 258:KAREKUY1, 260:KOMBARA, 261:KONANKOIRA, 262:KONONIBA, 263:MANI, 264:PATIARAKUY, 265:SAINT-JEAN, 266:SAINT-LOUIS, 267:SIMBADOUGOU, 268:SOA, 269:SOKORO, 270:TENOU, 271:THIA, 272:TOMBODOUGOU, 273:ZOUN, 275:BANTOMBO, 276:BOTTE, 277:KALLE, 278:KOURY, 279:LANFIERA-KOURA, 280:SIELA, 281:SONO, 282:SORO, 283:ZAMPANA, 285:sontorokuy, 286:yevedougou, 288:Homokuy, 289:Daborokuy, 290:Warkuy, 291:Koncoba, 292:Sayokuy, 293:Poe, 295:Werimbere, 296:Noumbere

LEGEND

Legend and structure of information in this file

Name of section

Enabling condition for this section

Question title

Type of question, scope

Answer options

Variable name

SECTION 5: OTHER INCOME SOURCES

E s4_other_sources_which.Contains(98)

Duis aute irure dolor in reprehenderit in voluptate velit esse cillum dolore eu fugiat nulla pariatur?

I This refers to family relations
E s3_time_other > 0
V1 s4_rel_leaders_which.Contains(98)
M1 Can not be itself
V2 (s3_time_other_breeding_advice <= (50 - s3_time_art_insem_advice)) ||s3_time_other_breeding_advice == 0
M2 This person is not in the list
F optioncode != s5_ignored_option_code

MULTI-SELECT
SCOPE: PREFILLED

s4_rel_leaders_other

01 ☐ Community animal health workers
02 ☐ Private
03 ☐ Government
04 ☐ Livestock keepers association
05 ☐ NGO

And 5 other [13]

Additional information:
"I" — Question instruction
"E" — Enabling condition
"V1" — Validation condition №1
"M1" — Message for validation №1
"F" — Filter in Categorical questions

Link to full set in appendix

Breadcrumbs

Type or roster

Roster Title

CHAPTER 3 IDENTIFICATION /
Roster: LEADER RELATION DETAILS
generated by fixed list:

01 Ward Livestock Officer
02 Village Livestock Officer
99 Other (specify)

List items
