# Supplementary material for: Feasibility, acceptability and validation of wearable devices for climate change and health research in the low-resource contexts of Burkina Faso and Kenya: Study protocol
Source: PLoS One. 2021 Sep 30;16(9):e0257170. doi: 10.1371/journal.pone.0257170 (PMC8483291; doi:10.1371/journal.pone.0257170)
Supplement: S2 File — (PDF) [file pone.0257170.s002.pdf]

Participant Information

Development and analysis for population health cohorts in Burkina Faso and Kenya

Principal Investigator:

- Prof. Till Bärnighausen, Heidelberg Institute of Global Health (HIGH), Universitaetsklinikum Heidelberg, Im Neuenheimer Feld 130.3, 69120 Heidelberg, Germany, Phone: +49 (0)6221 56-5637

Co-Investigators:

- Dr. Ali Sié, Centre de Recherche en Santé de Nouna (CRSN), Rue Namory KEÏTA, Nouna (Province dela Kossi), Burkina Faso, Phone: +226 20 53 70 43/55;
- Dr. Stephen Munga, Centre for Global Health Research (CGHR), Kenya Medical Research Institute (KEMRI), C/O Box 1578 – 40100, Kisumu, Kenya

---

Dear study participant,

we ask you for your participation in a study to improve health of individuals in health demographic surveillance (HDSS) sites, but also overall, by researching valid, reliable and socially and individually acceptable measurement instruments and infrastructure to conduct cutting-edge climate change and health research. To accomplish this goal, we will conduct research following two specific research objectives:

Objective 1: Elucidate the best approaches and pathways – and identify lessons learned – for the transition of existing standard HDSSs to HDSS that are enabled to conduct cutting- edge climate change and health research

Objective 2: Establish optimal approaches for novel sensor-based measurements for climate change and health research in HDSSs:

- Establish social and individual acceptability of novel sensors
- Determine reliability of novel sensors
- Determine long-term technical performance of novel sensors

The participation of you and your family in this study is completely voluntary.

The following text provides you with information on the background and procedure of the study.

Purpose of the Study

Climate change is becoming a major driver of ill health worldwide but in particular in sub-Saharan Africa. Few empirical studies, however, have focused on the impact of climate change or adaptation practices and interventions in poor communities in sub-Saharan Africa. A major reason for this scarcity of evidence is that the research infrastructures that support health research in sub-Saharan Africa – in particular HDSS are not equipped to investigate many of the most pressing research needs in climate change and health. Through this project, we aim to contribute the methodological insights on how to build a successful HDSS where climate change and health research can be as easily and productively carried out as other types of empirical population-based research. Furthermore, through focused methods studies, we aim to establish the acceptability, reliability, and durability of the next-generation of measurement instruments for this type of research – sensors for homes and for individuals.

For this reason, we want to find answers in our study on how to establish and how to best equip existing HDSSs worldwide with valid, reliable and socially and individually acceptable measurement instruments and infrastructure to conduct cutting-edge climate change and health research. To accomplish this goal, we will conduct *methods research*, while at the same time supporting the data collection that is needed for the other research teams that are part of this consortium.

The results of this study will help to understand how climate-ready data collection within HDSS can improve the overall health of adults in rural sub-Saharan Africa, especially with regard to climate change. This knowledge is needed to inform decision makers in your health system about strategies for adaptation and mitigation of climate-change induced health risks.

Course of the Study

Preceding the study, you are asked to read this participant information and to sign the informed consent form. All data and samples which we collect in this study will be treated confidentially and will be pseudonymised. This means that only code numbers and no personal names will be used when analyzing the data. The present program consists of 3 main parts.

Part 1: Mixed-methods case study

We will organize in-depth interviews (IDIs), involved actors for both HDSS (the directors of the two HDSS: 2x n=1, scientists 2x n=5, community leaders 2x n=3, and HDSS participants 2x n=6). In the first stage of the IDIs, we will elucidate best practices for existing HDSS of the INDEPTH network for climate change and health research, based on our lessons learned we will provide guidance and a concrete roadmap for implementing the necessary measurements and infrastructure for climate change and health research in an HDSS setting.

Furthermore, technological diaries serve to understand the processes and subjective experiences of implementing the novel measurements and infrastructure, such as weather stations, sensors, remotely sensed data etc., through the lenses of the involved actors at the two HDSS – fieldwork supervisors, technical support staff and fieldworkers. We will ask the different actors that play important roles in establishing and using the novel measurements and infrastructure to record their subjective impressions and their lived experiences regarding these technological changes. The diary is implemented offline on a tablet for mobile usage and online on a web-based platform with the software SurveySolutions (diary is structured to increase coherence of collected data, software allows to capture pictures) and is primarily targeted at implementers and maintainers of the study that comprise fieldworkers and supervisors (2x n=10), as well as technical support staff (2x n=5).

Part 2: Sensor acceptability and desirability

We will establish the acceptability and desirability of the different sensors using a survey that we will ask participants in this study to respond to once they have used the sensors for one week. For each human-based sensor, we will recruit 20 participants for our study from a representative sample of the HDSS communities in Burkina Faso and Kenya, reaching a total of 120 individual participants (2x locations * 3x sensors * 20 participants per location and sensor). For the three human-based sensors – the Tucky wearable thermometer (a patch), the Withings Pulse HR (a wristband), and the Kenzen patch – we will ask questions about the perceived ease of wear, comfort during sleep, comfort during daytime activities, satisfaction with the sensor placement, and reasons for non-wear We will use 5-point Likert-type scales and open-ended questions to elicit quantitative information on sensor acceptability. Besides, we will conduct individual IDIs starting with open-ended questions about the experience wearing and using one of the sensors. These open-ended questions will be followed by a number of probes regarding different dimensions of acceptability and desirability. In particular, we will probe how the experience of wearing and using the sensors could be improved in the future.

In addition to the human-based sensors, a questionnaire about sensor acceptability and desirability will ask study participants living in the homes that participate in the randomized controlled trial of the cool roof intervention (a total of 600 homes, and an estimated of 1,800 adults). The iButton DS1923 will be placed in the main living room/bedroom area of the houses. The iButton will be installed on an interior wall, about 1.5 meters above floor level (at adult head height, but above child head height, to reduce the chance of contact). We will ask questions about the aesthetic value of the home-based sensor (the iButton DS1923), the ease of use, the acceptability of repeated visits for iButton data download and electricity recharging. In 20 of these 1,800 adults, we will follow these initial interviews with an invitation to participate in IDIs on sensor acceptability and desirability. The IDIs will be translated and transcribed.

Part 3: Sensor reliability

Following the study of acceptability and desirability, we will again recruit a total of 2x3x20 = 120 representative adults living in the CRSN/Nouna and the KEMRI/Kisumu surveillance areas. In this study, we will ask them to wear two

sensors on the same body location (on the right-hand side vs. on the left-hand side). Similarly, we will place second iButton DS1923 sensors in 20 homes, in which the randomized controlled trial of cool roof effectiveness takes place. In all cases, we will measure the respective variables for a period of two weeks. The results of this study will shed insight on the technical feasibility based on the technological performance of the temperature and humidity sensor iButton DS1921G within selected households in the CRSN/Nouna HDSS.

Your pseudonymised data (i.e., only with a code number) will be transferred and will be analysed by the coordinating institution of the study. That is:

- Prof. Till Bärnighausen, Heidelberg Institute of Global Health (HIGH), Universitaetsklinikum Heidelberg, Im Neuenheimer Feld 130.3, 69120 Heidelberg, Germany, Phone: +49 (0) 6221 56-5637
- Dr. Ali Sié, Centre de Recherche en Santé de Nouna (CRSN), Rue Namory KEÏTA, Nouna (Province dela Kossi), Burkina Faso, Phone: +226 20 53 70 43/55;
- Dr. Stephen Munga, Centre for Global Health Research (CGHR), Kenya Medical Research Institute (KEMRI), C/O Box 1578 – 40100, Kisumu, Kenya

Potential Risks

Some participants can feel that the following is unpleasant or a burden:

- the interview takes too long;
- measuring body temperature and other vital data with a patch on the body;
- the pressure by the wrist-worn fitness tracker for measuring vital data;
- the notification of findings which, e.g., suggest certain health conditions.

There will be no invasive procedures, such as blood collection.

Potential Benefit

Likely, you and your family benefit from participating in this study, as part of the study comprises human-based sensors which measure vital data which may provide insights or indicate trends on certain aspects of your health performance. We will provide adequate, local treatment, in case poor health conditions are unveiled as part of the study. Moreover, the information derived from this study can help that this study translates into practice in your country as a tool to reduce the effects of climate change on health and to adapt to the foreseeable health problems that weather changes will create.

Data Protection

By signing the informed consent form, you declare that you agree that the principal investigator and his team members collect and process your personal data for the purpose of the above-mentioned analyses. Personal data are, e.g., name, birth date, address, health and vital data, amongst others. The purpose of storing and analyzing data is to measure the effect of the program and to understand how it has worked. The principal investigator will use your personal data for purposes of administration and study conduct. The data – after having assigned a pseudonym

– is for purposes of research in the field of climate change and health, and for statistical analysis. The code key – which allows bringing together your personal data with your identity – is accessible only for the principal investigators and his study team members. Using this code key, we will contact you by letter latest 5 years after your study participation to invite you to a follow-up examination. The code key will be destroyed after ten years.

The principal investigator passes your pseudonymized data on to the cooperation partners of the study who perform further statistical analyses.

You have the right of disclosure by the principal investigator of all personal data concerning you. You also have the right of correction of incorrect personal data. In these cases, please contact the principal investigator. You find his address and phone number below. **At any time**, you can cancel your agreement to participate in this study and/or your agreement to processing your data collected for this study. You can also request deletion of your data. Please also note that the (anonymous) results of this study may be published in scientific literature or other scientific contexts (i.e. conferences), in a form which does not allow any conclusions on your person.

Honorarium and costs

By participating in this study, there will be no costs for you involved.

Insurance

For formal reasons, we point out that no specific insurance has been contracted for study participants. Rather, the principal investigator is insured against liability claims which arise from culpable performance by the employer's liability insurance.

Voluntariness

Participation in this study is voluntary. You have the right – without giving reasons – not to participate in this study, to cancel your given consent, to cancel your given consent to the analysis of your data and samples, and to terminate your study participation without affecting your standard medical care in any way.

Contact for Questions

In case you have further questions, please contact your physician. Moreover, you can contact the principal investigator anytime for issues regarding the study:

Principal investigators and contacts:

- Prof. Dr. Dr. Till Bärnighausen, Heidelberg Institute of Global Health (HIGH), Universitaetsklinikum Heidelberg, Im Neuenheimer Feld 130.3, 69120 Heidelberg, Germany, Phone: +49 (0) 6221 56-5637
- Dr. Ali Sié, Centre de Recherche en Santé de Nouna (CRSN), Rue Namory KEÏTA, Nouna (Province de la Kossi), Burkina Faso, Phone: +226 (0) 20 53 70 43/55
- Dr. Stephen Munga, Centre for Global Health Research (CGHR), Kenya Medical Research Institute (KEMRI), C/O Box 1578 – 40100, Kisumu, Kenya

Heidelberg, 23rd April 2019

Prof. Dr. Dr. Till Bärnighausen
Principal Investigator
