# Supplementary material for: Feasibility, acceptability and validation of wearable devices for climate change and health research in the low-resource contexts of Burkina Faso and Kenya: Study protocol
Source: PLoS One. 2021 Sep 30;16(9):e0257170. doi: 10.1371/journal.pone.0257170 (PMC8483291; doi:10.1371/journal.pone.0257170)
Supplement: Attachment — Submitted filename: Response_to_Reviewers.pdf [file pone.0257170.s003.pdf]

Response to Reviewers

Dear Reviewers,

we would like to thank you for your comments and helpful suggestions to our manuscript. Accordingly, we have made amendments to the manuscript and describe our amendments with respect to the reviewers' comments in the following.

Reviewer #1: This is an interesting manuscript that comprises a study protocol for the purpose of conducting a climate change and health research in low-resource areas using wearable devices. Overall, the protocol looks fine and the expected results would be valuable.
I don't have major objections to the manuscript. I do have the following small issues for the authors to consider:

1. I suggest the authors to consider is it necessary to monitor all the devices to see if some are not working properly (e.g., if some of them are under direct sunshine and the battery got burned) and what to do if malfunctions do occur.

→ Many thanks for this comment. We do take up hardware reliability as part of this study and have added the following section to the manuscript, which we hope clarifies this point a bit further, p.8: "We will investigate the feasibility and acceptability with a Likert-scaled questionnaire that covers also demographic items: 13 items addressing feasibility with a strong focus on hardware reliability (i.e., are the devices functioning well in the sub-Saharan climatic conditions), and 29 items addressing acceptance (see S1 File for the questionnaire). If the wearable devices fail or we have data outages, we shall keep track of them in order to gain a quantitative overview of the types of failures and sources of data disruptions."

2. I'm not sure if (regular) incentives are necessary to provide to the participants to finish the study.

→ Indeed, study participants receive an adequate reimbursement (according to local standards). We have added on p.16 the following: "The study participants are reimbursed for their time commitment in the study and the type and scope are subject to local HDSS guidelines."

3. I'm not sure if the data would be enough for the authors to use deep learning models for analysis.

→ Again, many thanks for this comment. Indeed, this analysis is targeted for the subsequent one-year study that is planned to follow this study. We did not specify this well in the manuscript and after consideration have removed this paragraph, as it seems to be overburdening the current study protocol, which focus on feasibility, acceptability and validity.

Reviewer #2: This manuscript presents the study protocol to investigate the acceptability, feasibility and validity of some wearable devices to study a sub-Saharan Africa population subjected to heat stress. The presentation of the protocol is well described and the number of subjects who will be enrolled seems to be compatible with significant statistical power for the subsequent analysis.
The issue is certainly important considering the problem of climate change that we are experiencing in this era.

Perhaps it would be appropriate to specify the method of sterilization of the instrumentation supplied in the study also in light of the problems of Covid 19.

→ Many thanks for this comment, which covers an important aspect. We have specified the sterilization in light of COVID-19 in the manuscript, p.10/11: "To decrease the likelihood to transmit infectious diseases like the Coronavirus disease (Covid-19), each study nurse and community field worker is equipped with a hygiene package comprising face masks, disinfectant spray, clean wipes and hand disinfectant. The wearable devices are first rinsed with water, then sprayed with disinfectant, and wiped clean. A protocol that details this process is handed out to each study nurse and field worker."

A likely critical point within the various physiological parameters that will be monitored is the measurement of blood pressure, which is only foreseen at the time of the meeting with the nurse. This parameter will certainly not give information on the relative trend of blood pressure so it could be useful if a measurement within 24 hours of the subjects enrolled could be useful to be related to the environmental climatic characteristics.

→ Yes, we agree that a 24hour measurement would be of great help to correlate this with weather characteristics. However, after consideration, we have removed the blood pressure measurement for this study, as we primarily want to focus on the feasibility, acceptability and reliability of the wearable devices and adding blood pressure at this point in time may only add further complications instead of insights. We will consider your remark for our one-year study that is to follow this study and we will aim for a 24-hour measurement to get higher granularity of individuals response to local weather events with regards to a blood pressure measurement.

Considering also a possible purpose of the study which could be to evaluate the cardiovascular response to heat stress, it is also asked to specify how the environmental climatic parameters will be detected during the enrollment period of the subjects.

→ We have detailed specifics on the ten local weather stations that we have setup as part of this research unit in Kenya and Burkina Faso, p.8/9: "As part of this study, we have installed ten fully automated weather stations with the same configuration in the Nouna HDSS (5x) and in the Siaya HDSS (5x) that spatially cover the whole HDSS area. As a follow-up study to this feasibility, acceptability and validation study, it is planned to generate wearable measurements in a one-year study to account for seasonality and investigate whether weather (WBGT) has an effect on individual behavior and health (climate-sensitive disease, as available within the Nouna and Siaya HDSS). The local weather stations that have been setup as part of this research unit cover the continuous measurements of precipitation, wind speed, wind direction, radiation, temperature, and humidity, as well as WBGT. Seasonality has no bearing on the feasibility, acceptability, or validation of the wearable measurement in this study, hence it is not taken into consideration."
